# PhenoLink - a web-tool for linking phenotype to ~omics data for bacteria: application to gene-trait matching for *Lactobacillus plantarum *strains

**DOI:** 10.1186/1471-2164-13-170

**Published:** 2012-05-04

**Authors:** Jumamurat R Bayjanov, Douwe Molenaar, Vesela Tzeneva, Roland J Siezen, Sacha A F T van Hijum

**Affiliations:** 1 Centre for Molecular and Biomolecular Informatics, Radboud University Nijmegen Medical Centre, PO Box 9101, Nijmegen, The Netherlands; 2 Netherlands Bioinformatics Centre, 260 NBIC, P.O. Box 9101, Nijmegen 6500 HB, The Netherlands; 3 TI Food and Nutrition, P.O. Box 557, Wageningen 6700 AN, The Netherlands; 4 Kluyver Centre for Genomics of Industrial Fermentation, NIZO food research, P.O. Box 20, Ede 6710 BA, The Netherlands; 5 Systems Bioinformatics IBIVU, Free University of Amsterdam, Amsterdam 1081HV, The Netherlands

## Abstract

**Background:**

Linking phenotypes to high-throughput molecular biology information generated by ~omics technologies allows revealing cellular mechanisms underlying an organism's phenotype. ~Omics datasets are often very large and noisy with many features (e.g., genes, metabolite abundances). Thus, associating phenotypes to ~omics data requires an approach that is robust to noise and can handle large and diverse data sets.

**Results:**

We developed a web-tool PhenoLink (http://bamics2.cmbi.ru.nl/websoftware/phenolink/) that links phenotype to ~omics data sets using well-established as well new techniques. PhenoLink imputes missing values and preprocesses input data (i) to decrease inherent noise in the data and (ii) to counterbalance pitfalls of the Random Forest algorithm, on which feature (e.g., gene) selection is based. Preprocessed data is used in feature (e.g., gene) selection to identify relations to phenotypes. We applied PhenoLink to identify gene-phenotype relations based on the presence/absence of 2847 genes in 42 *Lactobacillus plantarum *strains and phenotypic measurements of these strains in several experimental conditions, including growth on sugars and nitrogen-dioxide production. Genes were ranked based on their importance (predictive value) to correctly predict the phenotype of a given strain. In addition to known gene to phenotype relations we also found novel relations.

**Conclusions:**

PhenoLink is an easily accessible web-tool to facilitate identifying relations from large and often noisy phenotype and ~omics datasets. Visualization of links to phenotypes offered in PhenoLink allows prioritizing links, finding relations between features, finding relations between phenotypes, and identifying outliers in phenotype data. PhenoLink can be used to uncover phenotype links to a multitude of ~omics data, e.g., gene presence/absence (determined by e.g.: CGH or next-generation sequencing), gene expression (determined by e.g.: microarrays or RNA-seq), or metabolite abundance (determined by e.g.: GC-MS).

## Background

The phenotype of an organism is governed by the interplay between genome content and cellular regulatory mechanisms. Recent high-throughput technologies such as microarrays, next-generation sequencing, RNA-seq, proteomics and metabolomics have the potential to profile presence, expression, and/or abundance of components involved in these regulatory mechanisms. These technologies assess simultaneously thousands of features (e.g.: gene presence, gene expression, metabolite abundance) generating large and often noisy data. The inherent noisiness results, for instance, in inaccurate genotype calling and/or inaccurate phenotypic measurements. Additionally, these features could have intrinsic relations (e.g.: correlation), which makes identifying links to phenotypes a non-trivial task.

Several methods have been devised to identify genetic links to phenotypes, which include a) correlating variation in SNP presence to phenotypes [1], b) correlating large-scale prokaryotic genomic data, obtained by integrating data from various sources like pathways and protein domains, with their phenotypes [2], c) combining closely related quantitative traits to identify genetic markers that jointly affect (a subset of) these traits [3], d) selecting gene sets based on their expression levels under different experimental conditions and then building phenotype-specific gene networks [4]. Though these methods allow finding relations between features and phenotypes, correlation based methods (a, b and c) are not suitable for finding partial relations, i.e. a feature that is important only for a subset of samples of a certain phenotype. Classification algorithms might capture such relations. In addition, ~omics data sets often have many more features (e.g. genes) than samples (e.g. strains). Classifying such data sets leads to the small *n *(e.g., dozens of observations) large *p *(e.g., thousands of features) problem, where estimating the true contribution of a feature becomes more difficult. ~Omics data also needs to be preprocessed for highly correlated features, as the contribution of such features in classification would be wrongly estimated in tree-based classifiers [5], and for features with homogeneous values across all observations as they decrease classification accuracy [6]. Additionally, in some experiments an imbalanced phenotype distribution could occur when most observations are of the same dominant phenotype. Many classification algorithms favor dominant phenotypes resulting in a biased classification [7], in turn resulting in fewer and weaker relations identified between features and minority phenotypes. Thus, to decrease noise both ~omics and phenotype data should be preprocessed before using these data in classification.

Identifying links to phenotypes from ~omics data is an essential part of an association analysis, yet prioritizing important links is often difficult without adequate visualization. Furthermore, some of the links could be spurious due to methodological reasons (e.g.: misclassification of a sample) and/or technological reasons (e.g.: cross hybridization of probes resulting in wrong genotyping). Therefore, visualization that integrates different information sources in a single figure such as networks [2] or color encoded figures allow identifying gene to phenotype links more straight-forward. Therefore, we encoded different levels of gene to phenotype or gene to strain relationships with color codes. In summary, determining links to phenotype(s) from large data sets requires an approach that is robust against noise, handles imbalanced phenotype data and provides a comprehensive yet general visualization of links to all phenotypes.

We developed a method, PhenoLink, that facilitates associating phenotypes to ~omics data (e.g.: gene presence/absence), is robust against noise, and decreases imbalance in phenotype data by a bagging procedure. The Random Forest classification algorithm is extensively used in ~omics data analysis because it is less resource-intensive than many classification algorithms (e.g.: Bayesian algorithms), it makes no implicit assumption regarding data properties and it is specifically suited to deal with the small *n *large *p *problem due to the use of many weak classifiers (see below). We use Random Forest to identify features that are relevant for phenotypes. The identified links are visualized to allow better mining of relations between phenotype and ~omics data. PhenoLink was implemented as a web-tool, which was applied to identify relations between genes (presence/absence) and phenotypes (sugar utilization and nitrogen-dioxide production) of 42 *Lactobacillus plantarum *strains. Although *L. plantarum *strain WCFS1 has been extensively studied in the past [8], we identified novel gene-to-phenotype relations in addition to already known ones.

## Results

### PhenoLink algorithm

PhenoLink is a web-tool developed to link phenotypes to ~omics data. We use the Random Forest classification algorithm [9], which builds an ensemble of decision trees, to find relations between features derived from ~omics data (e.g. gene presence/absence) and phenotypic readouts. Imbalance in phenotype data is decreased by bagging, and only one of the highly correlated and none of the homogeneous features [10] in ~omics data is used in classification. Based on the contribution score, only relevant features are used in subsequent round(s) of classification. Iterative feature selection allows the identification of only relevant relations between features and phenotypes.

### Applying PhenoLink to *L. plantarum *genotype and phenotype data

We used PhenoLink to identify genes that are important for phenotypes of 42 *L. plantarum *strains (see Additional file 1). The genotype data consists of the presence/absence of 2847 genes in these strains as determined by CGH arrays. The phenotype data contain measurements of these strains for sugar utilization (49 different sugars and control), and nitrogen-dioxide production (see Table 1). From 51 phenotypic experiments only 12 were usable in association analysis (see Methods).

**Table 1:** Phenotypic measurements of strains

Medium enriched with	Phenotypes^a^
D-arabitol	Yes (7), Maybe^b ^(11), No (21)
D-melezitose	Yes (34), No (5)
D-raffinose	Yes (33), Maybe (1), No(5)
D-sorbitol	Yes (35), No (4)
D-turanose	Yes (32), No (7)
Glycerol	Maybe (4), No (35)
K-gluconate	Yes (26), Maybe (9), No (4)
L-Arabinose	Yes (26), Maybe (3), No (10)
L-Rhamnose	Yes (6), Maybe (8), No (25)
Methyl-.d-glucopyranoside	Yes (8), Maybe (1), No (30)
Methyl-.d-mannopyranoside	Yes (27), Maybe (1), No (12)
Nitrogen-dioxide production	Yes (6), No (36)

**a**: Numbers in parenthesis show number of strains with given phenotype, for instance there are 32 strains that could grow on sugar D-turanose and 7 strains that could not grow on this sugar. Phenotype and ~omics data are available at the web address of PhenoLink.

**b**: For some strains phenotypes could not be determined accurately resulting in an ambiguous phenotype "Maybe". Using the web-interface of PhenoLink such phenotypes can be discarded from the association analysis (see Methods and User's Guide available at PhenoLink's web page).

Once homogeneous genes were removed only 610 genes remained and of these 271 remained after eliminating all but one of the highly-correlated genes (see Methods). The default variance of 5% leads to discarding all features that have different values in at most two strains for the *L. plantarum *dataset (see threshold guide available at PhenoLink website). Decrease in phenotype imbalance using bagging (see Methods) often allowed classifying four additional phenotypes. Though there were no big differences in classification accuracy for dominant phenotypes with or without bagging, phenotypes with the fewest strains mostly had a classification accuracy less than 30% and some of them having 0% classification accuracy without bagging. Bagging increased classification accuracy for some of these phenotypes up to 69%. There was not a significant decrease in median classification accuracy of all accurately classified phenotypes (less than a 1% decrease; see also the threshold guide). Visualizing identified relations allowed capturing genes and gene clusters that are related to single or multiple phenotypes, which are described below.

### Identifying novel and known links to phenotypes

**Figure 1:**
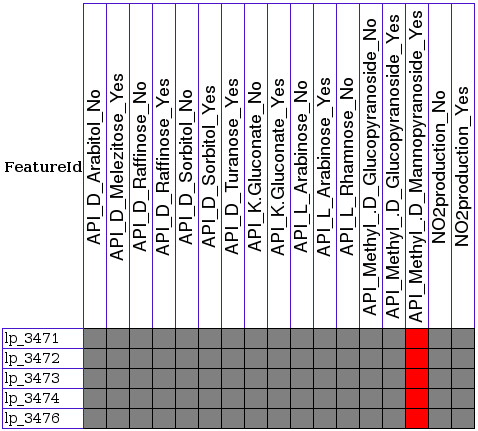
**Genes related to growth on Methyl-d-mannopyranoside**. Relations of genes to growth on Methyl-d-mannopyranoside as visualized by PhenoLink. For gene annotations see Additional file 2. Color codes are explained in Figure 4. Colors of surrounding genes can be seen by running PhenoLink with the same configurations used here.

**Figure 2:**
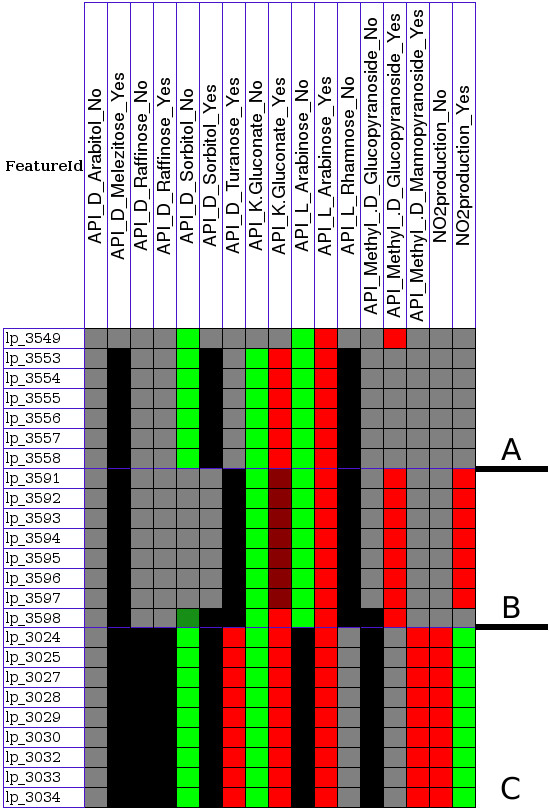
**Gene clusters related to utilization of multiple sugars**. Relations of L-arabinose (A) and L-rhamnose (B) gene clusters to utilization of multiple sugars as visualized by PhenoLink. A cluster of 9 genes is related to multiple sugars and nitrogen-dioxide production (C). Partial relations between genes and growth on sorbitol are shown in Additional file 3. For gene annotations see Additional file 2. Color codes are explained in Figure 4. Colors of surrounding genes can be seen by running PhenoLink with the same configurations used here.

Only a few gene clusters were found that relate to a single phenotype. For instance, a cluster of five genes (lp_3471-lp_3476) is found as important for Methyl-d-mannopyranoside utilization (see Figure 1). In contrast, we found several gene clusters that are related to multiple phenotypes, and these include novel and well-described gene clusters such as those for L-arabinose [11] (Figure 2) and L-rhamnose metabolism [12] (Figure 2). Most of the genes were related to growth on sugars encode enzyme (24.2%), transport (17.7%) or regulatory (15.1%) functions. Many genes were related to multiple phenotypes (81% of all genes linked to phenotypes), which is partly due to the fact that some transporters and enzymes are promiscuous and can utilize several structurally related sugars. However, more importantly this demonstrates that for a manifestation of any phenotype often more than a single gene is important.

A very large gene cluster of 28 genes was found to be related to nitrate reduction and nitrogen dioxide production (see Additional file 4 and Additional file 2) by *L. plantarum *strains; this gene cluster was already experimentally validated to be involved in nitrogen dioxide production [13]. Another gene cluster containing 9 genes (see Figure 2) was found to be related to utilization of multiple sugars and to absence of nitrogen-dioxide production.

### Comparison of PhenoLink and correlation-based methods

For all usable *L. plantarum *phenotypic tests, genes that were found to be related to phenotypes were the same using both Pearson and Spearman correlation metrics (see Methods). On average 69% of these genes were also found by PhenoLink. However, on average 37% of the genes that were found by PhenoLink to be related to any phenotype were found by correlation. The remaining genes identified by PhenoLink could not be found by these two conventional correlation methods, mostly due to the partial relations to phenotypes or they are related to phenotypes present in fewer strains, and PhenoLink effectively deals with such phenotype imbalance by using bagging. Nevertheless, most of the clear relations between phenotypes and genes can be identified by both methods. For phenotypes of strains assessed by three API tests (D-arabitol, D-raffinose and Methyl-glucopyranoside) no gene was found to be related by any of the correlation methods. For the remaining phenotype data on average 89 and 131 genes were found for phenotypes of each experiment, respectively by the correlation methods and by PhenoLink. However, some relations that were identified by correlation methods could not be identified by PhenoLink, because correlation-based methods often found genes that were only related to the dominant phenotype regardless of the gene's presence/absence values in strains of other phenotypes. Such biased relations are largely decreased by bagging and these genes are less likely to get higher contribution scores by the Random Forest algorithm as they could not be used to categorize strains of different phenotypes accurately. Most of the genes that were only identified by correlation methods have hypothetical, regulatory or transport and binding functions. Though the genes that were only identified by PhenoLink have similar functions, there were many protein encoding genes which were not identified by correlation. Both methods identified some phage proteins or transposases to be related to phenotypes and using a p-value of 0.01 decreases such spurious links. However as a consequence it would be impossible to identify any relation for many phenotypes based on the correlation-based methods.

### Applying PhenoLink to *S. pneumonia *genomic array footprinting based gene essentiality data

We also used PhenoLink with *S. pneumoniae *gene essentiality data to identify 10 genes that were found to be significant from a genomic array footprinting experiment [14] and that were experimentally proven to be significantly attenuated during meningitis infection. A ratio-based analysis of signals at initial time point (t_0_) and at time point *n *was used by the authors to prioritize genes affected by transposon insertions (see Methods). A ratio-based analysis is in general more suited for data from microarray experiments where two dyes were used, due to that signals from both dyes of the same hybridization share many systematic errors occurring in microarray experiments, e.g., position on the slide surface and scanning effects [10]. We analyzed this noisy dataset with PhenoLink using a default classification-based analysis. The classification was based on signals for the respective time points. As this dataset is very noisy, a signal based classification is expected to be less suited than a ratio-based analysis. The PhenoLink based classification accuracy was high for all classes (see Methods) except for experiments at time point 3 hours. Therefore, experimental results at time point 3 hour were not used in PhenoLink; however, the full dataset is available at the PhenoLink's web-site. We found 7 (see Additional file 5) of the validated 10 genes (shown in Figure four in the publication of Molzen et. al.) by using PhenoLink with similar parameters as it was used for *L. plantarum *dataset (see Methods). Neighbours for some of these genes were also found and, as expected, some of them are in the same operon. Additionally, we also found a few new genes to be related to different time-points Identified operon members and new targets are now being evaluated by a co-author of the genomic array footprinting study (Dr. P. Burghout).

## Discussion

Linking phenotypes to ~omics datasets is difficult due to size and noisiness of the data and lack of easily accessible tools that can (i) handle large and noisy data, (ii) find links to phenotypes and (iii) visualize links. We developed a web-tool - PhenoLink - to identify links to phenotypes using classification-based feature selection (in this study genes). The presence/absence of 2847 genes in 42 *L. plantarum *strains and phenotypes of these strains was used in PhenoLink to identify links to phenotypes assessed in 51 experiments. We tried different visualization techniques such as graph and tree structures for enhanced visualization of identified relations. In visualization as much as possible information should be embedded in a single figure, while still it should be easy to interpret. Visualization of identified links using different colours for each relationship type allowed capturing relations not only between genes and phenotypes, but also relations among genes and among phenotypes. Additionally, visualization allowed identifying partial relations between genes and phenotypes (shown in black colours), where different genes are essential for certain strains of a phenotype. For instance, among correctly classified polysaccharide (D-melezitose, D-turanose and D- raffinose) utilization experiments, only for D-raffinose additional polysaccharide biosynthesis genes (lp_1197, lp_1198 and lp_1199) were found to be related (see Additional file 6). All strains that can't grow on raffinose lack these genes and most of the growing strains have either these genes or other polysaccharide biosynthesis genes (lp_1216-lp_1227) (see Additional file 7). Possibly the growing strains can utilize raffinose degradation product for polysaccharide biosynthesis.

The *L. plantarum *strains used in this study often showed similar phenotypes, or some of them had ambiguously defined phenotypes such as "Maybe" due to either mild expression of a trait or possibly experimental error. Strains with this phenotype are, as expected, often misclassified. Therefore in this study, we discarded all strains with this phenotype; however it can be configured in our web tool to include them. Using ambiguous phenotypes in certain cases could be beneficial to validate input data as strains with similar gene content should have similar or the same phenotype. PhenoLink can be configured to generate classification accuracy plots for each experiment (see Additional file 8), which shows how accurately strains were classified. Reasons for consistent misclassification of strains are: (i) ambiguous or wrong phenotype, (ii) noisy ~omics data, and/or (iii) these strains could belong to a minority phenotype (see Methods).

In the present example, the presence/absence of genes was determined based on hybridization to an array containing probes to only a single reference strain (WCFS1) and its three plasmids. Because *L. plantarum *is a versatile species living in various environments, the gene content of many of these strains will be in part different from that of WCFS1 [8]. Strain NIZO2776 is exceptional, as it was always misclassified to be not growing on the sugar L-arabinose (see Additional file 8). Based on CGH data, 16.6% of the genes of strain WCFS1 are predicted to be absent in strain NIZO2776 [8]; however other strains that lack even more WCFS1 genes have correctly been classified. Probably this strain either does not grow on L-arabinose (wrong phenotyping) or it uses different sets of genes to grow on L-arabinose, which differ too much in sequence compared to WCFS1 genes in order to be detected by CGH. Pan-genome arrays specifying probes based on the genomic content of multiple strains and plasmids within the same species, should provide a better estimate of species-level genomic divergence. However, cross-hybridization of probes is the general disadvantage of the microarray technology, which leads to inaccurate gene calling. With prices decreasing continuously, next-generation sequencing techniques are becoming better alternatives due to their accuracy and recall of new or divergent genes, that using microarrays would have been missed. Gene presence/absence determined by sequencing would allow PhenoLink to determine links to phenotypes more accurately.

PhenoLink allows decreasing huge combinations of possible experimental tests by pruning input data and prioritizing identified links. Though many phenotypes (more than 55%) were classified with accuracy above 80%, we used a 60% classification accuracy cutoff to accommodate noise in input data such as wrong gene calling or imbalance in phenotype data. Identifying partial relations is inherently difficult even with classification-based association analysis. Thus such findings, which are visualized in a black colour, should first be corroborated with available literature and/or databases before performing follow-up lab-experiments.

PhenoLink allows finding links to many phenotypes of several strains. The input data should contain information about at least a few strains (default of 4) with at least two different phenotypes (totaling 8 strains). However, most of the public data sets often lack either ~omics or phenotype data. Most of the ~omics and/or phenotype data sets are from studies of only a few strains, posing the small sample size problem preventing their use in PhenoLink, and yet many others had a phenotype imbalance problem [15,16]. In this study, we describe the use of PhenoLink on two different datasets: (i) *Lactobacillus plantarum *genotype and phenotype data and (ii) *Streptococcus pneumoniae *gene essentiality data. These datasets are publicly available (see PhenoLink website).

In PhenoLink, redundant and noisy features are removed before association analysis. Therefore an increase in the number of features would not increase proportionally the total run-time. We tested the increase in PhenoLink's run-time as a function of an increase in the number of features. To this end we created two datasets by increasing total number of features for 42 *L. plantarum *strains from 2847 to 5000 and 10000. An increase in the number of features exponentially increased PhenoLink run-time. One has to note that this is likely due to that unlike with the actual *L. plantarum *data, most features in the randomly generated data had very high variances and were not often correlated. This in turn substantially increased the number of features used in classification, and PhenoLink's run-time.

We developed a web-tool - PhenoLink - to link phenotypes to ~omics data. It is a flexible and versatile tool. PhenoLink can be used to effectively prioritize links from different ~omics datasets, such as genotype, transcriptome, metabolome, proteome to phenotypes. It is a tool with enhanced visualization of links to phenotypes, is more accurate than correlation-based method and less resource-intensive than Bayesian-based methods. It has already been used in several studies to identify leads to phenotypes from diverse sets of ~omics data such as genotype, transcriptome and metabolome data. Thus, PhenoLink facilitates screening large ~omics and phenotype data sets, allowing to effectively capture known relations to phenotypes as well as novel relations.

## Conclusions

Linking phenotypes to large ~omics data sets is essential for generating leads to understand the underlying mechanism of a phenotype. Often such analysis is hindered by the scale of data and lack of easy-to-use tools. We present an easily-accessible web-tool, PhenoLink incorporates well-studied techniques such as feature selection, bagging, removing redundant or noisy features and a feature selection stability criterion into the single tool. Using an enhanced visualization, this tool can be used to address different problems with different data types and data sizes. It preprocesses input data to decrease noise and uses classification-based feature selection to accurately find features that are related to phenotypes. It identifies links to phenotypes more accurately than correlation-based methods, efficiently handles large data sets and is robust against noise [17]. Visualization allows quick identification of relations (i) between features and phenotypes, (ii) among features, (iii) among phenotypes, (iv) features and samples, which use different feature sets to exhibit the same phenotype, and also (v) outliers in phenotype data, which would allow detecting possible experimental errors. Identified links might be used to improve feature annotations (in this study gene annotation) in selected cases with limited experimental validation. PhenoLink therefore allows researchers to quickly screen large data sets for new leads to phenotype associations.

## Methods

### The PhenoLink algorithm

**Figure 3:**
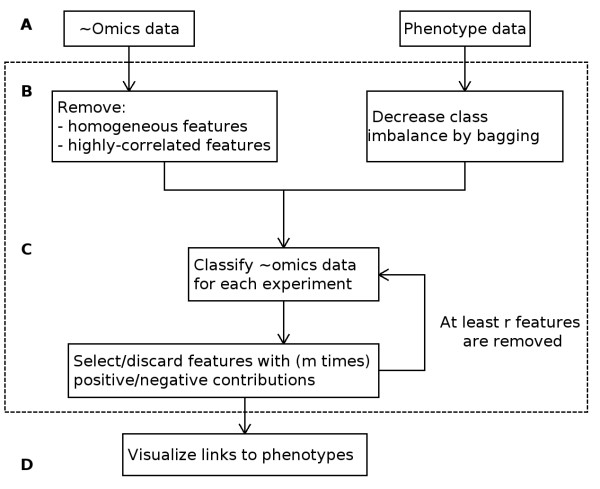
**Flow diagram for a web-tool PhenoLink**. Default values of *m *and *r *are 3 and 5 respectively (see Methods section for more information).

Our phenotype to ~omics association algorithm consists of three steps (Figure 3): (i) data preprocessing; (ii) feature selection using classification and (iii) visual representation of links to phenotypes. Below follows a description of each step.

### Data preprocessing

#### Removing missing values

As example, we used the presence/absence of genes in 42 *Lactobacillus plantarum *strains, determined from comparative genome hybridization (CGH) data (see below), to relate to strain phenotypes: (i) growth in various sugar-rich environments and (ii) nitrogen-dioxide production. The phenotype (e.g.: growth or non-growth) of some strains could in some cases not be determined due to technical reasons. This results in missing values for particular phenotypic measurements for some strains, which were not used in the analysis of that phenotype. Missing values in ~omics data can be imputed by PhenoLink using a method supplied in the *randomForest *R package [18], though for this study we did not have missing values in the genotype data.

#### Removing homogeneous features

Features (e.g. genes) with little variance (below the default of 5% therefore having virtually identical presence patterns) across all strains are removed to reduce redundancy and complexity of the genotype data. Removing such genes in our case leads to a 30% decrease in genotype data size, and hence decrease in noise [6], and a significant decrease in computational time.

#### Removing highly correlated features

The Random Forest algorithm builds many decision trees by randomly sampling a subset of the samples (in this study strains) and randomly sampling a small subset of features (in this study genes). The best split in this set is used as the split of a given node in a tree. Once one of the highly-correlated features is used to split a node in a tree, other correlated features would get a much lower importance. Because importance of correlated features is biased towards their selection order in tree building [5], only one of the highly-correlated features, based on Pearson (for linear relations) and Spearman (for non-linear relations) correlation metrics, is used. This leads to improved classification accuracy and prevents dilution of contribution score for a feature in classification [19]. If the correlation score of any feature-pair is above predefined cutoffs for Pearson (default value of 0.98) and Spearman (default value of 0.95) correlations, they are considered to have (almost) identical presence/absence patterns: often these genes could be co-inherited, e.g. in the same operon(s). After features that link to phenotype(s) are selected by classification (see below), the discarded and highly-correlated features to the selected features (e.g. operon members) are added to the PhenoLink output not to miss any relations.

### Feature selection using classification

#### Classification

Classifying ~omics data with respect to phenotype data often leads to the small *n *large *p *problem, with many more features (in this study genes) than samples (in this study strains). In this study, 2847 genes from comparative genome hybridization (CGH) analysis and phenotype data for 42 *L. plantarum *strains were used. We used the Random Forest algorithm [9] implemented in the R package *randomForest *(version 4.5-28) [18] for the gene-phenotype matching as (i) it is difficult to overtrain, (ii) it is non-parametric - no implicit assumption about any parameter of input data is made, (iii) it generates for each phenotype a classification error, which represents the percentage of strains with a particular phenotype have been misclassified, and (iv) it assesses a contribution score (i.e. the local importance of a gene) for each gene to correctly classify a strain to belong to given phenotype (class). The Random Forest algorithm builds an ensemble of decision trees, where each tree is trained by randomly sampling both ~omics and phenotype data. Using contribution score of genes for strains with the same phenotype, subset(s) of strains can be identified to which different genes are related.

#### Decreasing class imbalance by bagging

Although the Random Forest algorithm is very suited for classifying ~omics datasets, like many classification algorithms, it has a bias towards majority classes (dominant phenotypes) [7,20]. These are phenotypes which are found in (far) more strains than other phenotype(s) [7,21]. Solutions include several bagging techniques like over-sampling, down-sizing and multiple down-sizing to decrease differences in class sizes while keeping similar data distribution compared to the original data [20,21]. Bagging allows identifying partial relations between features and phenotypes while effectively dealing with dominant phenotypes. We devised a different bagging technique to guarantee that all strains of the dominant phenotype are used at least *l *(default values of 10) times in classification. In order to create a bag, all strains of the smallest phenotype, which has the fewest strains, are selected and a larger set of strains are selected from each of the remaining phenotypes. The size of the strain set was empirically defined as the two times the total number of strains of the smallest phenotype (see threshold guide), which can be changed in the web-interface. Sampling continues until the remaining strains of a phenotype are insufficient to create a full bag. In this case all remaining strains of a phenotype are selected and strains that were used in previous bags are sampled randomly. This procedure is continued for *l *times. We term this bagging technique as multiple-covering, because all strains of the dominant phenotype are covered at least *l *times. Each bag is classified separately after which the feature contribution scores and classification errors of each bag are averaged. Both bagging techniques, multiple down-sizing and multiple-covering, are available in PhenoLink. The latter should only be used for very large datasets, because with the multiple down-sizing technique many bags need to be created to ensure all instances were selected sufficiently and classifying many bags of large data would increase total run-time rapidly. In this study we used the former by setting parameter *l *to 100 and bagging should always be used, because bags would only be created if in the supplied phenotype data the instances per phenotype are highly disproportionally distributed.

#### Iterative removal of insignificant features

Although almost 90% of features are discarded before classification by removing homogeneous and highly-correlated genes (see above), for the *L. plantarum *dataset still almost 300 genes remain. Data from each phenotypic experiment is classified, using the Random Forest algorithm, *m *times (in this study 3) with the same genotype data. Genes that consistently have positive or negative contribution scores for at least three strains of the same phenotype are selected or discarded. Additionally the percentage of instances of a given phenotype can also be used to discard features that are likely to be irrelevant. This circumvents identifying a feature as relevant, which can by chance have a positive contribution score for 3 instances of a phenotype with many instances. Iteration of the feature selection process for *m *times improves feature selection stability, i.e. selects only the most relevant features [22]. After eliminating features, classification is performed again until fewer than *r *(in this study 5) features are removed.

#### Adding correlated features

Only a phenotype of which 60% of strains are correctly classified by the Random Forest algorithm is considered in further analysis. This accuracy cutoff level of 60% allows to capture even weak relations often resulting from noise in input data such as wrong gene calling. After recursive elimination of non-informative features for each phenotype that is classified with sufficient accuracy, the top *t *(default of 50) features are selected based on their phenotype importance, which is the sum of the feature's contribution score to classify each strain of this phenotype. In order to capture most of the genotype-phenotype relations, we also add features that are highly correlated to any of these top *t *features using the above-mentioned two correlation metrics. Added features assume the phenotype importance of a feature to which they are correlated.

#### Categorizing continuous values in ~omics data

In visualizing identified relations to a phenotype, occurrence information of a feature in strains of a particular phenotype is integrated with the phenotype importance (see next section). Therefore only for visualization of feature-phenotype relations, continuous values in ~omics data (e.g.: gene expression data) are categorized into two groups by assigning 0 (absent) to values below the predefined cutoff and 1 (present) to values greater than or equal to this cutoff. The cutoff value should be specified in the web-interface; by default the average of maximum and minimum values is used as in this study.

#### Categorizing continuous values in phenotype data

The Random Forest algorithm generates useful classification-related information, which allows in-depth analysis and better visualization of identified relations. Classification, unlike regression, allows identifying common features for groups of strains that belong to the same class (phenotype). Additionally visualizing relations between each phenotype measurement and its related features would lead to many targets, and therefore create large figures, which are difficult to interpret. Therefore in PhenoLink, continuous phenotypic measurements are grouped by binning to perform classification analysis instead of regression. The default value of 3 bins could be adjusted depending on data type and number of instances (see user's guide and threshold guide).

### Visual representation of links to phenotypes

**Figure 4:**
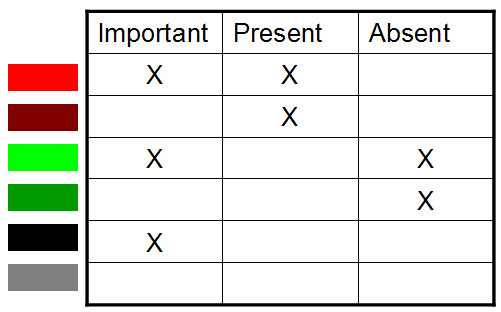
**Integration of gene significance with its presence/absence in different strains**. A feature (in this study a gene) that is found to be important to separate strains of different phenotypes is assumed important. Present (for the majority of strains): feature is present in at least *p *percent (default of 75%) of strains for a given phenotype. Absent (for the majority of strains): feature is absent in at least 75% of strains of a given phenotype. Remaining genes are present in a subset of strains.

In order to better visualize links to phenotypes the contribution score (estimated by the Random Forest algorithm) of a feature is merged with its observed value (in this study presence/absence of a gene) in strains of all accurately classified phenotypes. Such visualization facilitates identifying relations between features, relations between features and single/multiple phenotypes, and relations between phenotypes. In order to visualize genotype-phenotype relations for all phenotypes together, relations are visualized in 6 different categories each encoded with a different color. Therefore, we define the presence and absence of a feature for a phenotype according to the following criteria: i) a feature is assumed as sufficiently present if it is present in at least 75% of strains that have this particular phenotype; ii) a feature is assumed as sufficiently absent if it is absent in at least 75% of strains that have this particular phenotype; iii) otherwise a feature is present in a subset of strains with a particular phenotype (see Figure 4). We use a default cutoff of 75%, which can be altered in the web-interface, for effective separation of stronger feature-phenotype relations from partial relations. For enhanced screening of the relations, the presence/absence of a feature for a phenotype is merged with its phenotype importance resulting in 6 different levels of feature-phenotype relations (see Figure 4). However, we use the presence/absence of features as determined from CGH data in visualizing links to phenotypes for each experiment separately to identify strain-level importance of a feature.

### Comparison of Random Forest and correlation-based feature selection methods

Many studies use correlation between features and phenotypes [1-3] to identify feature to phenotype relations. We used Pearson's (linear relations) and Spearman's (non-linear relations) correlation metrics to find features that are highly correlated to phenotypes. Features that have high positive or negative correlation to phenotypes get lower p-values and p-values are adjusted for multiple testing resulting in the false discovery rate using the "fdr" method in R with the p.adjust function [23]. For each correlation metric all features with adjusted p-value 0.05 or smaller were selected.

### Categorizing identified relations

We divided identified relations into three groups: (i) incorrect relations, (ii) confirming previous observations and (iii) novel relations. The predicted relations that were contradicted in public literature or deemed very unlikely based on other information such as gene annotation and phenotype information were assumed to be incorrectly predicted relations. Incorrect predictions could be due to data e.g.: noise, sample size, method e.g.: less bags, lower accuracy threshold, or both. Other relations were assumed as (potentially) correct predictions. If correct predictions were not described elsewhere, based on (i) a literature search, (ii) the STRING database [24] or (iii) NCBI's Protein Clusters [25] databases, we assumed them to be novel. Identified relations that were already published were assumed correct confirming predictions. Here, we describe only confirming and novel predictions. However the described gene-phenotype relations were mostly selected using gene annotation information, which could lead to ignoring some possibly novel relations. Therefore all relations encoded in bright red and bright green colours (see Figure 4) could be submitted for follow-up analyses.

### Experimental data

#### Strains

We used data of 42 *L. plantarum *strains (see Additional file 1) for genotype-phenotype association analysis [8].

#### Phenotype data

The analytical profile index (API) test was used with 50 different sugars as substrates to identify growing and non-growing *L. plantarum *strains on these sugars [8]. Phenotype data could only be used in association analysis if it meets these two criteria: (i) in an experiment all strains cannot have the same phenotype and (ii) there are at least *k *(default value of 4) strains with any phenotype. Using the first criterion, phenotype information of 31 experiments was removed (*e.g*. all strains grow or do not grow on a given sugar) leaving growth information on 19 different substrates. Among these, phenotype information for a total of 11 substrates met the second criterion (see Table 1) [8]. In addition to API tests, we also used information on nitrogen dioxide production [13] by these strains (see Table 1 and also Additional file 9). Both genotype and phenotype data sets can be downloaded from the web address of PhenoLink. Frequently, strains with an ambiguous phenotype like 'Maybe' have been misclassified, thereby decreasing classification accuracy. We therefore did not use strains with this phenotype in association analysis; however, by default all strains are used.

#### Microarray design and CGH analysis

The presence or absence of genes in the selected 42 *L. plantarum *strains (see Additional file 1) was determined using comparative genome hybridization (CGH) microarrays. A total of 8555 60-mer nucleotide probes targeted 2805 annotated open reading frames (ORFs) of the chromosome and 42 ORFs of three plasmids of *L. plantarum *WCFS1. On average each ORF was targeted by three probes evenly distributed over its entire sequence and each probe was present in duplicate on the array. The microarray design was deposited in the Gene Expression Omnibus (GEO) database and is available under accession number GPL5874 [8].

#### Normalization of microarrays

Many of the arrays contained considerable spatial bias as values of M = log_2_(I_sample_/I_reference_) or A = log_2_(I_sample _* I_reference_) displayed clear spatial patterns on the array (see Additional file 10). To remove this spatial bias, M and A were each separately corrected using the kernel-smoothing function from the "fields" package in R applying a normal kernel [26]. An example of the results of such normalization is shown in the upper two rows of Additional file 10. Subsequently, to correct dye intensity bias, a loess fit [27] was performed on data points that had an M-value differing by at most 1 from the median of all M-values. This span includes most data corresponding to probes for genes present in the query strain. The loess fitting is shown in Additional file 10 in the lower left graph.

#### Probe-sequence and gene presence/absence calling

The presence or absence of a complementary sequence for a certain probe in the sample strain was based on the corrected M (ratio of WCFS1 to sample strain) values. The M-values for all probes were plotted in a histogram (see lower-centre graph in Additional file 10). The histograms showed two peaks, (i) a major one near M = 0: probes with a sample fluorescence close to that of WCFS1, and (ii) a minor peak at (very low) M-values: probes that signify absence of the target sequence in the sample strain. A threshold value for M that lies at the minimum between the two peaks of the histogram, which was determined for each array separately, was used to derive presence or absence from the M value of a given probe. This minimum was determined by calculating a smoothed numeric derivative, using a Lanczos differentiator [28] with 21-bin window on the M-value histogram divided in 400 bins, and by locating the position, below M = −0.5, where it traversed from values below to values above zero (see lower central graph in Additional file 10). This M-value corresponds to a value closest to the minimum between the peaks.

The presence or absence of a gene from the reference strain in a sample strain was decided by majority vote of presence/absence calls of the probes with sequences corresponding to those of the gene. Most genes were represented by three probes with different sequences (see Microarray Design section). In few cases (around 0.2% of cases among all hybridizations) an equal number of probes voted for and against the presence of the gene, in which case they were assumed to be absent.

#### S. pneumoniae gene essentiality data

We used gene essentiality data based on a transposon mutant library of *S. pneumoniae *[14] to test PhenoLink. This dataset consists of 45 dual-channel microarrays, which assess signal differences of 2087 open reading frames (ORFs) of *S. pneumoniae *TIGR4 strain (see Molzen et. al. for more information). Array analysis was performed at 3 different time points with intervals 3, 9 and 15 hours. The microarray data can be obtained from the NCBI Gene Expression Omnibus database (http://www.ncbi.nlm.nih.gov/geo/) with series accession number GSE21729. We classified the gene signals as function of different time points, different transposon libraries, different fluorescence channels (Cy3 and Cy5), and with different combinations of time points, libraries and channels. In total there were 6 different combinations (see PhenoLink's website for the datasets) and all were used in association analysis. We used Minomics to determine if relevant genes identified by PhenoLink are in the same operon [29].

### Software availability

PhenoLink is accessible at http://bamics2.cmbi.ru.nl/websoftware/phenolink/ and datasets that were used to demonstrate its applicability are available at this website as well as user and threshold guides. Source files and brief installation instructions of PhenoLink can be downloaded from http://trac.nbic.nl/phenolink.

## Competing interests

The authors declare that they have no competing interests.

## Authors' contributions

SvH, RJS conceived this study, JRB and SvH created the association analysis work-flow and web-tool, DM performed the CGH and microarray data analyses, VT performed sugar growth experiments, JRB/RJS/SvH performed data analyses, JB and SvH drafted the manuscript, All authors read and approved the manuscript.

## Supplementary Material

Additional file 1 **Table of *L. plantarum *strains used in genotype-phenotype matching analysis **[8]. Click here for file

Additional file 2 **Table containing functional annotations of genes related to single or multiple phenotypes**. Click here for file

Additional file 3 **Partial relations between genes and growth on sorbitol as shown in Figure 2**. Genes that were found as relevant for multiple phenotypes shown in Figure 2 were present in a subset of strains that grow on sorbitol (black). These genes are present in most (26 out of 35) of the growing strains and absent in all non-growing strains (bright green both in this figure and Figure 2). Click here for file

Additional file 4 **Genes related to nitrogen-dioxide production as visualized by PhenoLink **[13]. Click here for file

Additional file 5 **Table containing significantly attenuated genes during experimental meningitis **[14], **which were used to test performance of PhenoLink**. Click here for file

Additional file 6 **Polysaccharide biosynthesis genes related to multiple phenotypes as visualized by PhenoLink**. Part of polysaccharide biosynthesis cluster (lp_1197-lp_1199) was found to be related to only D-raffinose sugar utilization. For polysaccharides D-melezitose and D-turanose only other polysaccharide biosynthesis gene cluster (lp_1215-lp_1227) was found as relevant. Note that lp_1215 and lp_1216 both are glycosyltransferases, but lp_1215 was not found as relevant to none of the polysaccharide utilization tests. Annotations of these polysaccharide biosynthesis genes are given in Additional File 2. Click here for file

Additional file 7 **Polysaccharide biosynthesis genes found as relevant for strains used in D-raffinose utilization test**. Visualization of merging presence/absence and contribution score of polysaccharide biosynthesis genes for strains used in D-raffinose utilization test. Annotations of these genes are given in Additional File 2. Click here for file

Additional file 8 **A bar plot of classification accuracy per strain**. A bar plot showing number of times a strain have been correctly (black part of the bar) and incorrectly (gray part of the bar) classified by the Random Forest algorithm. Corresponding phenotypes ("Yes" for growth and "No" for no growth) of strains are shown as suffixes to strain names on the left side of the figure. For this figure phenotype data from L-arabinose utilization test was used. Click here for file

Additional file 9 **Table of *L. plantarum *strains ordered by nitrogen dioxide production levels**. Click here for file

Additional file 10 **Figure showing results of the normalization procedure of a CGH array**. The upper row shows the normalization of spatial bias for M =log(Isample/Ireference) and the middle row shows the normalization of spatial bias of A=log(Isample * Ireference). The left graphs in the upper two rows show the raw data, the middle two graphs show the kernel-smoothed results using a normal kernel, and the right graphs show the raw results corrected for spatial bias using the smoothed values from the middle graphs. The lower left graph shows a plot of the spatial bias corrected values of M and A for each probe on the array. The red line is a loess fit through the bulk of the data. The lower middle graph shows a histogram of spatial bias and loess-corrected M-values. The vertical red line is drawn at the position of the histogram with the highest M-value below -0.5 where the smoothed numeric derivative of the histogram was still negative (i.e. as close as possible to where it traverses the zero-line!). This boundary was used to define whether a probe signifies presence or absence of the targeting sequence (i.e. left or right of the red line). Finally, the lower right graph shows the spatial bias and loess-corrected M-A plot with probes classified as signaling presence (green) or absence (blue) of the targeting sequence. Click here for file
