# Supplementary figures and images for: PhenoLink - a web-tool for linking phenotype to ~omics data for bacteria: application to gene-trait matching for Lactobacillus plantarum strains
Source: BMC Genomics. 2012 May 4;13:170. doi: 10.1186/1471-2164-13-170 (PMC3366882; doi:10.1186/1471-2164-13-170)

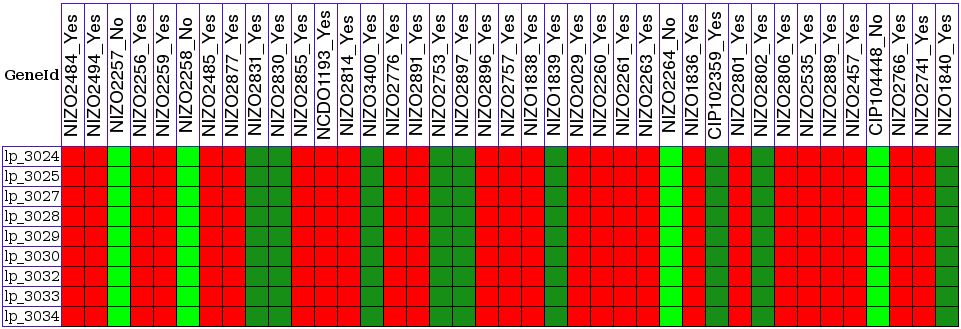

Supplement: Additional file 3 — Partial relations between genes and growth on sorbitol as shown in Figure 2. Genes that were found as relevant for multiple phenotypes shown in Figure 2 were present in a subset of strains that grow on sorbitol (black). These genes are present in most (26 out of 35) of the growing strains and absent in all non-growing strains (bright green both in this figure and Figure 2). [file 1471-2164-13-170-S3.PNG]

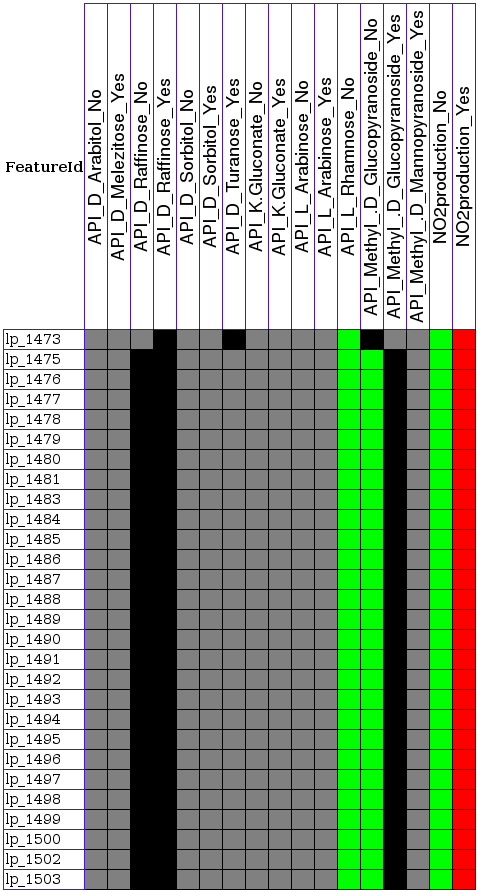

Supplement: Additional file 4 — Genes related to nitrogen-dioxide production as visualized by PhenoLink [13]. [file 1471-2164-13-170-S4.PNG]

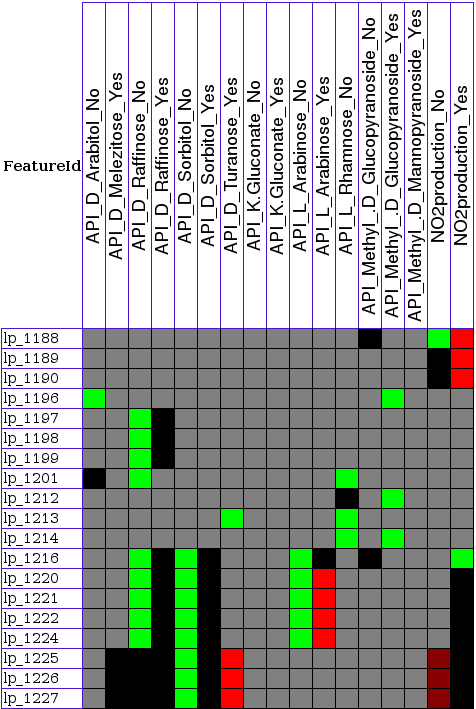

Supplement: Additional file 6 — Polysaccharide biosynthesis genes related to multiple phenotypes as visualized by PhenoLink. Part of polysaccharide biosynthesis cluster (lp_1197-lp_1199) was found to be related to only D-raffinose sugar utilization. For polysaccharides D-melezitose and D-turanose only other polysaccharide biosynthesis gene cluster (lp_1215-lp_1227) was found as relevant. Note that lp_1215 and lp_1216 both are glycosyltransferases, but lp_1215 was not found as relevant to none of the polysaccharide utilization tests. Annotations of these polysaccharide biosynthesis genes are given in Additional File 2. [file 1471-2164-13-170-S6.PNG]

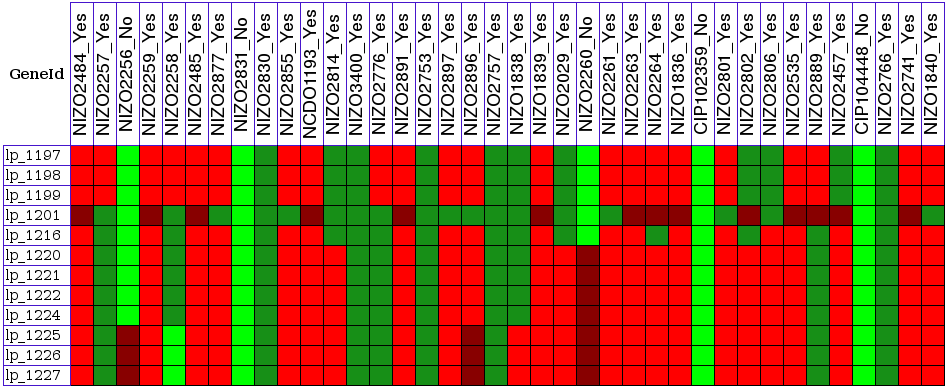

Supplement: Additional file 7 — Polysaccharide biosynthesis genes found as relevant for strains used in D-raffinose utilization test. Visualization of merging presence/absence and contribution score of polysaccharide biosynthesis genes for strains used in D-raffinose utilization test. Annotations of these genes are given in Additional File 2. [file 1471-2164-13-170-S7.PNG]

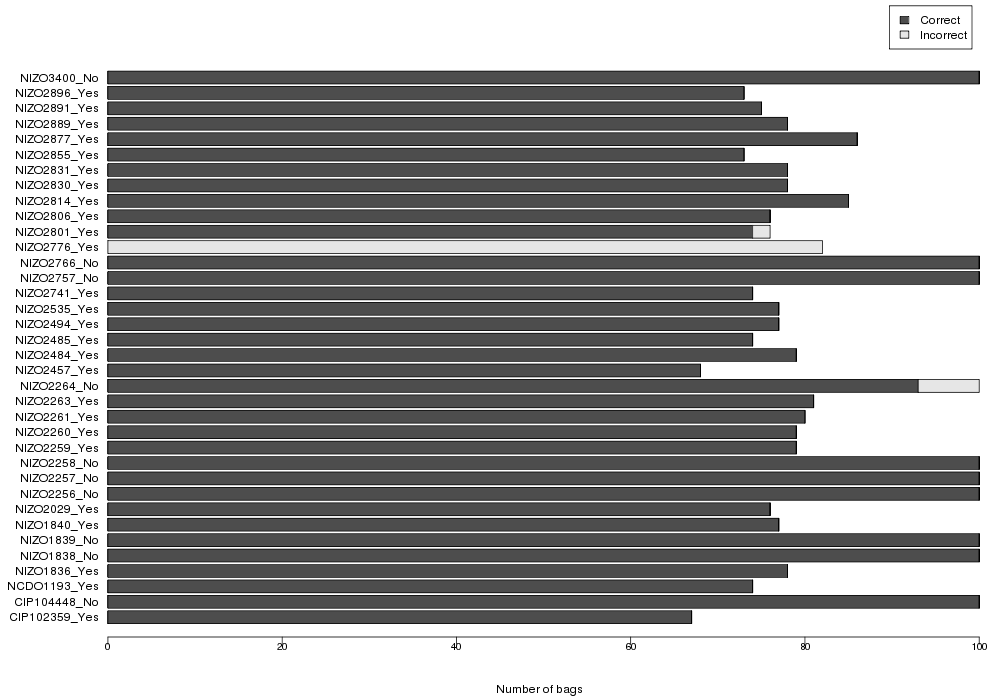

Supplement: Additional file 8 — A bar plot of classification accuracy per strain. A bar plot showing number of times a strain have been correctly (black part of the bar) and incorrectly (gray part of the bar) classified by the Random Forest algorithm. Corresponding phenotypes ("Yes" for growth and "No" for no growth) of strains are shown as suffixes to strain names on the left side of the figure. For this figure phenotype data from L-arabinose utilization test was used. [file 1471-2164-13-170-S8.PNG]

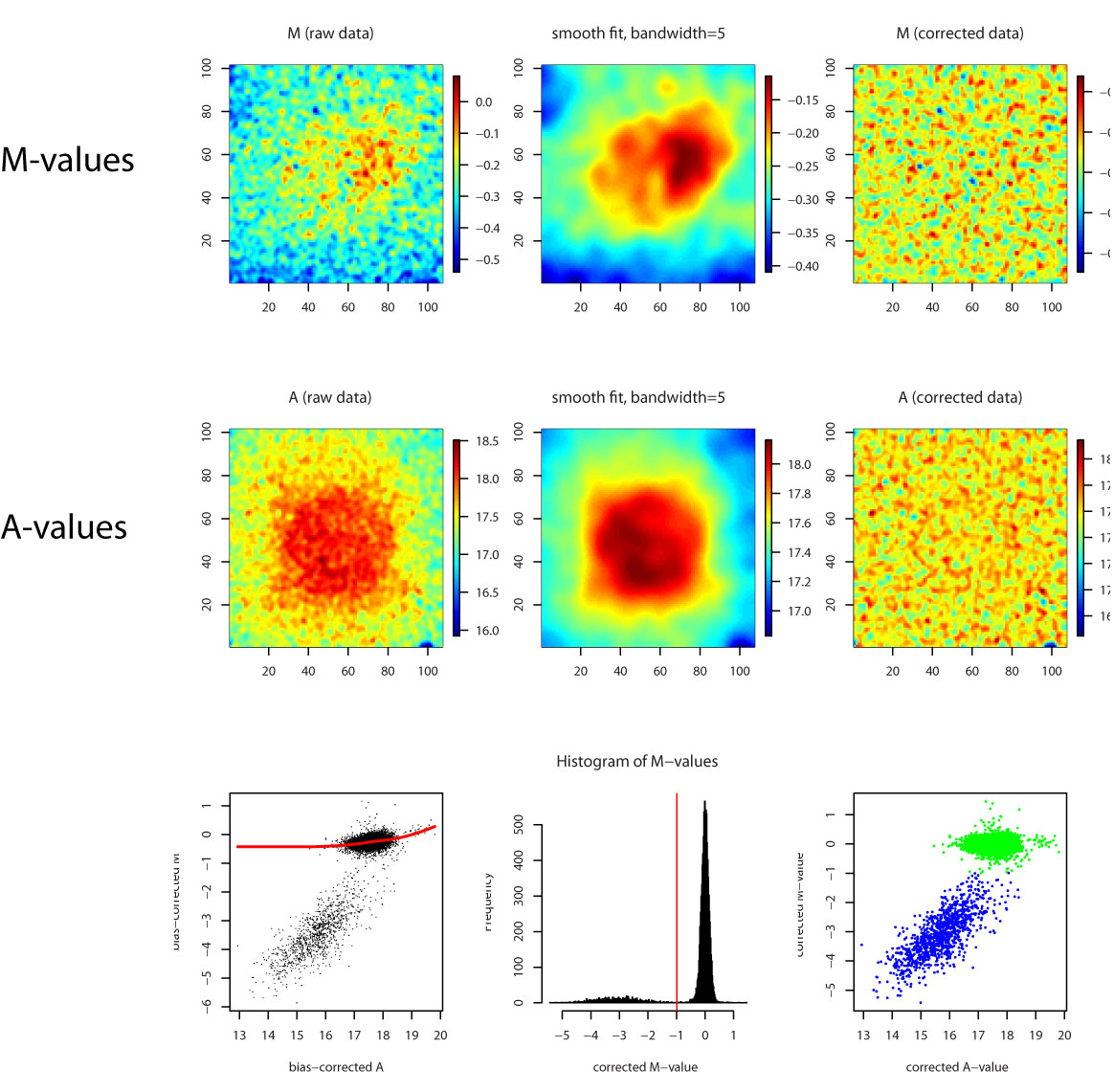

Supplement: Additional file 10 — Figure showing results of the normalization procedure of a CGH array. The upper row shows the normalization of spatial bias for M =log(Isample/Ireference) and the middle row shows the normalization of spatial bias of A=log(Isample * Ireference). The left graphs in the upper two rows show the raw data, the middle two graphs show the kernel-smoothed results using a normal kernel, and the right graphs show the raw results corrected for spatial bias using the smoothed values from the middle graphs. The lower left graph shows a plot of the spatial bias corrected values of M and A for each probe on the array. The red line is a loess fit through the bulk of the data. The lower middle graph shows a histogram of spatial bias and loess-corrected M-values. The vertical red line is drawn at the position of the histogram with the highest M-value below -0.5 where the smoothed numeric derivative of the histogram was still negative (i.e. as close as possible to where it traverses the zero-line!). This boundary was used to define whether a probe signifies presence or absence of the targeting sequence (i.e. left or right of the red line). Finally, the lower right graph shows the spatial bias and loess-corrected M-A plot with probes classified as signaling presence (green) or absence (blue) of the targeting sequence. [file 1471-2164-13-170-S10.PNG]
